# Transcranial focused ultrasound neuromodulation of the human primary motor cortex

**DOI:** 10.1038/s41598-018-28320-1

**Published:** 2018-07-03

**Authors:** Wynn Legon, Priya Bansal, Roman Tyshynsky, Leo Ai, Jerel K. Mueller

**Affiliations:** 1 Division of Physical Therapy and Division of Rehabilitation Science, Department of Rehabilitation Medicine, Medical School, University of Minnesota, Minneapolis, MN USA; 2 Department of Neuroscience, University of Minnesota, Minneapolis, MN USA; 3 Department of Neurosurgery, School of Medicine, University of Virginia, Charlottesville, VA United States

## Abstract

Transcranial focused ultrasound is an emerging form of non-invasive neuromodulation that uses acoustic energy to affect neuronal excitability. The effect of ultrasound on human motor cortical excitability and behavior is currently unknown. We apply ultrasound to the primary motor cortex in humans using a novel simultaneous transcranial ultrasound and magnetic stimulation paradigm that allows for concurrent and concentric ultrasound stimulation with transcranial magnetic stimulation (TMS). This allows for non-invasive inspection of the effect of ultrasound on motor neuronal excitability using the motor evoked potential (MEP). We test the effect of ultrasound on single pulse MEP recruitment curves and paired pulse protocols including short interval intracortical inhibition (SICI) and intracortical facilitation (ICF). In addition, we test the effect of ultrasound to motor cortex on a stimulus response reaction time task. Results show ultrasound inhibits the amplitude of single-pulse MEPs and attenuates intracortical facilitation but does not affect intracortical inhibition. Ultrasound also reduces reaction time on a simple stimulus response task. This is the first report of the effect of ultrasound on human motor cortical excitability and motor behavior and confirms previous results in the somatosensory cortex that ultrasound results in effective neuronal inhibition that confers a performance advantage.

## Introduction

Transcranial focused ultrasound (tFUS) is an innovative approach to noninvasive neuromodulation that uses focused mechanical energy to provide high spatial resolution targeting of distinct cortical areas^1^. Previous research has shown that tFUS modulates neuronal activity in mice^2–4^, rats^5^, rabbits^6^, sheep^7^, pigs^8^ and monkeys^9^. tFUS has also been demonstrated to be a safe and effective method for transient neuromodulation in human somatosensory cortex^1,10,11^, visual cortex^12^ and thalamus^13^. In Legon *et al*.^1^ we demonstrated tFUS to inhibit the primary somatosensory cortex and for this neuromodulation to be spatially specific. In addition, we found ultrasound to improve tactile discrimination performance. Follow-up studies in somatosensory cortex additionally showed ultrasound to elicit and augment tactile sensations in the hands of participants^10,11^. In visual cortex, low intensity focused ultrasound was demonstrated to elicit a blood-oxygen level dependent (BOLD) response in primary visual cortex and associated visual areas with reported phosphene elicitation^12^. More recently, we have demonstrated the ability of transcranial focused ultrasound to modulate sub-cortical areas of the human brain. tFUS directed at the thalamus resulted in an attenuation of somatosensory evoked potentials generated in the ventroposterior-lateral nucleus and serially connected cortical regions^13^. Here, we extend these findings to the human primary motor cortex (M1).

To assess the effect of ultrasound to M1 we have developed a novel method of simultaneous transcranial ultrasound and magnetic stimulation that pairs concurrent and concentric delivery of focused ultrasound with transcranial magnetic stimulation (TMS). This pairing is advantageous as it provides for assessment of ultrasound on established and well-understood TMS metrics like the motor evoked potential (MEP). In addition, it allows for a non-invasive examination of the effect of ultrasound on specific neuronal populations as different TMS methodologies (e.g. paired-pulse) have been demonstrated to preferentially affect different motor microcircuits^14^.

We test the effect of focused ultrasound to M1 on single pulse MEP recruitment curves, paired-pulse inter-stimulus intervals (1–15 msec) and on a simple stimulus response reaction time task. Based upon previous literature assessing the effect of tFUS on small and large animal motor cortex that has found ultrasound to elicit peripheral motor responses^2–7^, tFUS to human motor cortex could have excitatory effects and increase MEP amplitude; however, from our work in human somatosensory cortex we expect the overall effect to be for inhibition of motor cortical excitability.

Using different TMS methods such as short interval intracortical inhibition (SICI) and intra-cortical facilitation (ICF) that probe different motor micro-circuitry^15^ in addition to assessing a simple motor task we hope to gain a better understanding of the effect of tFUS in human motor cortex and the neuronal circuits that ultrasonic mechanical energy affects.

## Materials and Methods

### Subjects

All experiments were conducted with the approval of the University of Minnesota institutional review board and all experiments were performed in accordance with relevant guidelines and regulations. A total of 50 healthy volunteers; 16 men and 34 women, aged 19 to 38 years (22 ± 3.59 years) participated in the experiments. All subjects gave written informed consent and were financially compensated for participating. Subjects were screened for eligibility and confirmed to be physically and neurologically healthy with no history of neurological disorder. Additionally, subjects were cross-checked for known medication contraindications to other non-invasive neuromodulation techniques^16^.

### Simultaneous transcranial ultrasound and TMS

**Figure 1:**
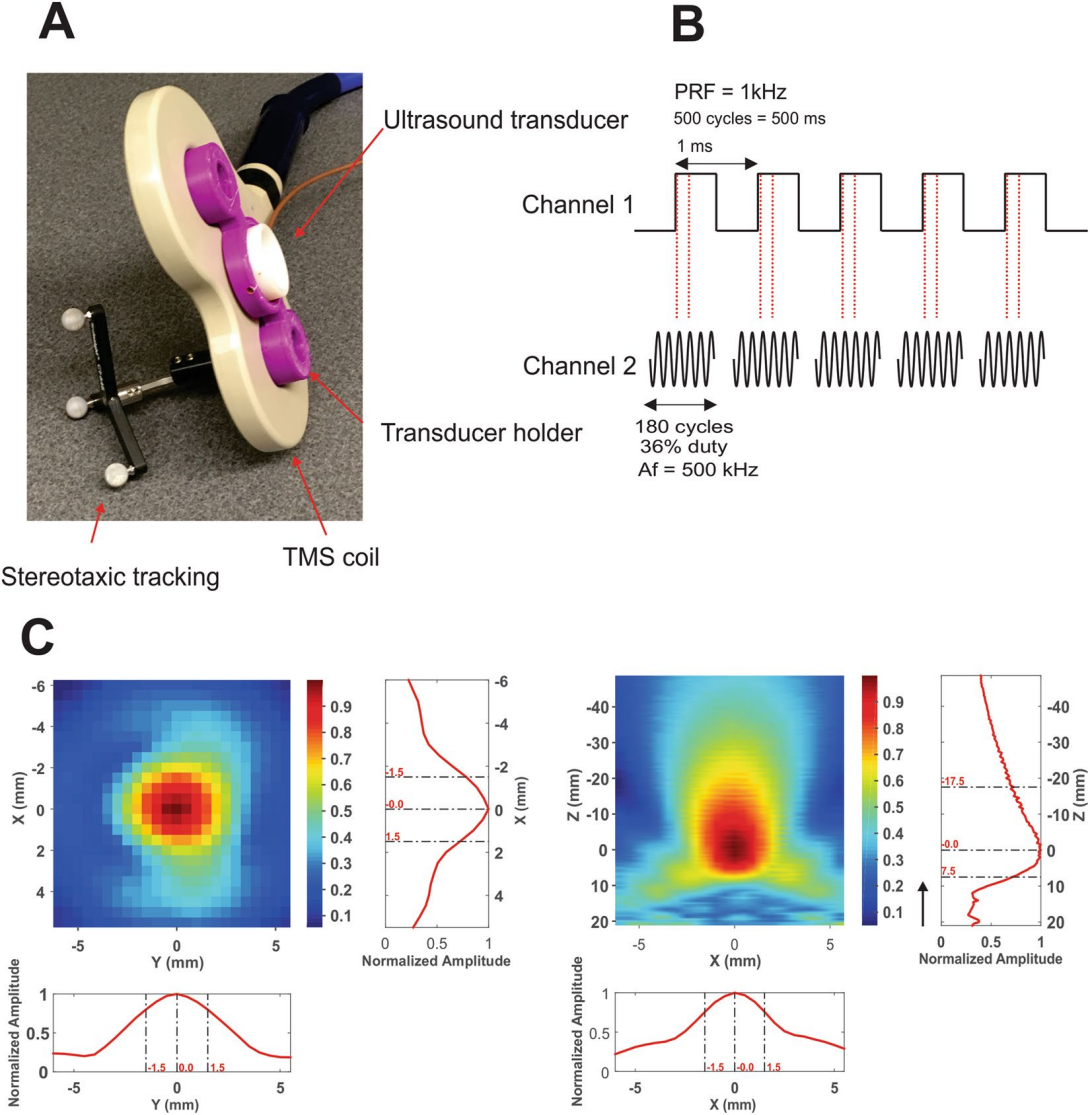
Transcranial ultrasound and magnetic stimulation. (**A**) Photograph of the Ultrasound/TMS device showing the TMS coil (beige), ultrasound transducer (white) and the holder (purple). Tracking bulbs are also visible and used to guide Ultrasound/TMS to a specific brain target using stereotaxic neuronavigation. (**B**) Ultrasound pulsing strategy. PRF = pulse repetition frequency; Af = acoustic frequency. (**C**) Pseudo-color and line free water plots of ultrasound pressure field. Scales are normalized to maximum pressure. Black arrow indicates direction of sonication.

The simultaneous application of ultrasound and TMS uses coil uses a standard commercial figure eight coil of two coplanar coil windings of equal size (double 70 mm alpha coil Magstim Inc., UK) to which a custom made low profile (1.25 cm height) single element 0.5 MHz focused ultrasound transducer^17^ is attached at the center of the coil intersection of the coil windings using a custom 3D printed holder (Fig. 1A). Initial testing was conducted to study the safety and feasibility of concurrent and concentric tFUS/TMS. Initial feasibility testing concentrated on the potential interaction of the two energy sources and potential damage or induction of current in the ultrasound transducer from the TMS magnetic field and for the ultrasound transducer to affect the magnetic field produced by the TMS coil and subsequent electric field produced in the head. To measure the effect of TMS on the resultant sound field we constructed a custom acoustic tank that separated the TMS coil from the water but left the transducer at center axis 1 mm from the face of the TMS coil. A hydrophone (HNR-0500 Onda Corp. Sunnyvale CA, USA) was placed in the tank at the measured focus of transducer. The ultrasound transducer used is a custom-made single element focused transducer fabricated as detailed in Kim *et al*.^17^. Acoustic frequency was 0.5 MHz transducer with a focal length of 22 mm, diameter of 36.5 mm and height of 12.5 mm. Testing consisted of 100 single pulses of 100% stimulator output TMS delivered at an inter-stimulus interval (ISI) of 8 seconds with and without concomitant ultrasound. The resultant TMS artifact and sound field were captured by the hydrophone in the test tank using test software and 3D stage (Precision Acoustics Dorset, UK). The TMS artifact observed by the hydrophone without the application of ultrasound was first recorded, followed by the pressure field from the US transducer (250 cycles) without TMS. Then, TMS was delivered concurrently with US at the same points to compare the pressures produced by the transducer with and without TMS. Traces of the TMS artifact alone were first subtracted from traces captured with simultaneous US and TMS and compared to the pressure from US alone. Next, we tested the effect of the US transducer on the magnetic field produced by the TMS coil. We measured the resultant magnetic vector potential from the center of the coil using a custom made magnetic probe made of two rectangular shaped windings of wires (1 cm^2^ surface area) that were oriented perpendicularly to each other^18^ and placed in plane with the face of the ultrasound transducer 1.25 cm above the face of the TMS coil. A piece of paper outlining a 3 × 3 cm grid was placed in plane with the face of the US transducer above the TMS coil as a guide for measurement points of the magnetic vector potential. Single pulse TMS was delivered at 100% stimulator output at an ISI of 8 seconds and recorded in the two axes of the probe at all points of the grid both with and without the ultrasound transducer present at the center of the TMS coil. Comparison of the magnetic vector potential measurements at each of the points with and without the US transducer were conducted and compared between the transducer and no transducer using a paired t-test.

To test the impact of the standoff height of the transducer on the induced electric field in the head from TMS, we modeled the 70 mm figure eight coil this using SIMNIBS 2.0 (www.simnibs.de). We modeled two conditions: a posterior to anterior current with the 70 mm figure eight coil tangential to the scalp over the primary motor cortex with handle posterior angled at 45 degrees from midline resting against the scalp, and the other condition kept all parameters the same except had the TMS coil offset from the scalp by 1.25 cm; the height of the transducer.

### tFUS waveform and delivery

For all experiments we used a custom-made single element focused transducer with a center frequency of 0.5 MHz, 30 mm aperture and 22 mm focal length. The pressure map in free water from this transducer is pictured in Fig. 1C. The calculated spatial peak pulse average intensity (I_sppa_) in free water was 17.12 W/cm^2^ with a corresponding spatial peak temporal average (I_spta_) of 6.16 W/cm^2^. The mechanical index was 0.9.

The waveform used was the same as previously described^1,19^. This waveform was generated using a two-channel 2-MHz function generator (BK Precision Instruments, CA, USA). Channel 1 was set to deliver tFUS at a pulse repetition frequency (PRF) at 1 kHz and channel 2 was set to drive the transducer at 500 kHz in burst mode while using channel 1 as the trigger for channel 2. Channel 2 was set to deliver 180 cycles per pulse, and channel 1 was set to deliver 500 pulses, resulting in a 500 msec duration. Channel 2 output was sent to a 100 W linear amplifier (2100 L Electronics & Innovation Ltd, NY, USA), with the output of the amplifier sent to the custom made, low profile tFUS transducer (Fig. 1B). For both the single pulse and paired pulsed experiments, ultrasound was time-locked to occur 100 msec prior to the TMS pulse. For all experiments, the ultrasound condition involved acoustically coupling the active face of the ultrasound transducer to the scalp at the pre-determined site (see stereotaxic neuronavigation). To achieve acoustic coupling to the head, the hair was parted to expose the scalp and ultrasound gel was used to keep the hair out of the way and ensure proper coupling with the ultrasound transducer. The transducer was also prepped with ultrasound gel on the surface that met the head, and was then placed on the exposed scalp as has been previously employed^1,19,20^. The sham condition involved turning over the transducer so that no ultrasound was delivered to the head, though contact was maintained ensuring equitable auditory artifact. This is important as in some cases, the piezoelectric element in the transducer produced a slightly audible buzzing. As such, participants were provided ear protection and did not report any sensible differences between sham and ultrasound for any of the experiments.

### Quantitative modeling of ultrasound wave propagation

A computational model was run to visualize and evaluate the wave propagation of tFUS across an example skull to determine the resultant intracranial beam profile and pressure map in the brain. The model was run using a magnetic resonance (MR) imaging and computerized tomography (CT) dataset taken from the Visible Human Project®^21^. The transducer was placed on the scalp site overlying the hand knob of the primary motor cortex. Simulations were performed using the k-Wave MATLAB toolbox^22^. Methods and details of the modeling parameters can be found in Mueller *et al*.^23^.

### Stereotaxic neuronavigation

Identification of all targeted areas for all experiments was aided and confirmed using a stereotaxic neuronavigation system (BrainSight, Rogue Research, Montreal, QUE, CAN) that was retrofitted to work with the TMS coil and the individual tFUS transducer used in the reaction time experiment (see Fig. 1A). Participants were fitted with infrared sensors to track head movement and individual fiducial head markers were used to align the participants’ head with a template brain in the neuronavigation system. The TMS coil and the ultrasound transducer (reaction time experiment) were also fitted with infrared markers allowing for detailed position of the coil or transducer relative to the participant head. This aided in initial targeting the primary motor cortex but more importantly ensured constant placement of the TMS coil and tFUS transducer trial to trial throughout the duration of all experiments. Target tolerance was set <= 1 mm from the original determined neuromodulation position.

### Electromyography (EMG)

For all experiments surface peripheral EMG was collected using surface adhesive electrodes (Medi-Trace® 530 series) in a belly tendon montage ground to the medial epicondyle of the ipsilateral humerus. Electrodes were additionally secured in place with tape to ensure good continuous contact and no movement. For the single and paired pulse experiments we recorded from the first dorsal interosseous (FDI) of the dominant hand. For the reaction time experiment we recorded from the dominant abductor pollis brevis (APB) as this was the muscle performing the task. EMG data was continuously recorded using a DC amplifier (Net Amps 400, Electrical Geodesics, Inc. Eugene, OR, USA) sampled at 1 kHz. Data was stored on a PC for later offline analysis. EMG analysis was performed using custom-made scripts written in Matlab (Mathworks Natick MA USA). The continuous data was first filtered forwards and backwards to avoid edge artifacts and to eliminate phase shifting using a 3^rd^ order Butterworth bandpass filter with cutoffs 5–200 Hz. The data was then epoched −50 to 100 msec around the onset of TMS delivery. Motor evoked potential (MEP) amplitudes were identified visually and quantified using the peak to peak method. An MEP was considered to be present if easily identifiable peaks were apparent above the noise floor of that trial in a 50 millisecond time window post TMS onset. Events where no MEP was identified were given an amplitude of zero and included in subsequent data analysis.

### Targeting of M1 hotspots

Targeting of M1 was similar for each experiment. For each participant, identification of the MEP ‘hotspot’ was determined by first setting the TMS stimulator output to 50% of stimulator maximum. The TMS coil (no ultrasound transducer) was positioned over the contralateral somatomotor region with the handle positioned backwards at 45 degrees from midline for a posterior to anterior induced current. Single pulses in sets of five (ISI = 10 seconds) were delivered and stimulator intensity adjusted between sets until MEPs at least 50 µV were elicited for three of the five stimulations. Subsequent target areas were tested spaced 1 cm anterior, posterior, lateral and medial to the initial target to verify the lowest possible stimulator output to meet threshold criteria. If one of these targets proved to have a lower threshold than the initially identified target, a new set of five single pulses was delivered to areas 1 cm anterior, posterior, lateral and medial to this new spot to ensure the lowest threshold of stimulation. This process was repeated until the site of lowest intensity was determined. Spacing of 1 cm was used as this was double the lateral spatial resolution of the ultrasound transducer used and thus provided spatially discrete (non-overlapping) sites for potential hotspot location. An additional step was necessary as the pairing of ultrasound with TMS produces a different resting motor threshold (RMT) as compared to the TMS coil alone (due to the standoff height of the ultrasound transducer). At the pre-determined hotspot as determined above, RMT for simultaneous ultrasound and TMS was determined using sets of ten stimuli (ISI = 10 seconds) and the stimulator output was set at a percent stimulator output that resulted in MEPs of at least 50 uV 50% of the time. In general, the Ultrasound/TMS RMT was about 20–30% higher than the RMT of the TMS coil alone. In cases where the Ultrasound/TMS RMT resulted in a stimulator output >100%, further study was not possible and the participant did not continue in the study.

### Experiment 1: Single-Pulse MEP recruitment curves

#### Subjects

A total of twelve individuals (4 male, 8 female, 23.4 ± 1.87 years) participated in this experiment. All were self-reported right hand dominant.

#### Experimental procedures

To generate recruitment curves, individual resting motor threshold for the dominant first dorsal interosseous (FDI) was determined as above for the Ultrasound/TMS coil and a stimulator output 20% below this value (rounded to the nearest 5%) was used as the starting value. For example, if a participant had a RMT of 73% stimulator output, their initial testing point would be 55% stimulator output (73–20 rounded to nearest 5%). Testing increments were conducted beginning at the above calculated starting stimulator intensity and increased in increments of 5% until 100% stimulator output was reached. A total of ten single pulses were collected for each stimulator output at an ISI of 10 seconds. Prior to collection of the recruitment curve, a baseline condition (no ultrasound, but with 1.25 cm standoff) was collected of 10 single pulses spaced 10 seconds apart at individual Ultrasound/TMS RMT. This procedure was completed for both tFUS and sham conditions, counterbalanced across participants. Stimulation time was approximately 15–20 minutes for each condition, and total experiment time including setup was approximately 2 hours.

#### Analysis

To test for significant differences in the MEP recruitment curves between sham and Ultrasound/TMS neuromodulation a two-way repeated measures analysis of variance (ANOVA) was performed with main factors MODULATION (real, sham) and TMS INTENSITY (75%, 80% 85%, 90%, 95%, 100%). Only data points from stimulator intensities 75–100% were included as these were the stimulator intensities that generally produced non-zero quantifiable MEPs across participants that could be appropriately statistically tested. Main effects were examined using Tukey-Kramer post-hoc testing.

### Experiment 2: Paired-Pulse MEP

#### Subjects

A total of ten individuals (3 male, 7 female, 22 ± 3.24 years) participated in this experiment. All were self-reported right hand dominant.

#### Experimental procedures

The paired pulse technique for this study was adapted from^24^ where a sub-threshold conditioning stimulus is followed by a supra-threshold test stimulus delivered through the same coil at a given latency. Prior to paired-pulse testing, Ultrasound/TMS RMT was established so as to determine the 80% and 120% TMS stimulator outputs for paired pulse testing. A baseline condition was then completed as ten single pulses at 120% Ultrasound/TMS RMT spaced at 10 seconds. After baseline collection, the Ultrasound/TMS device was positioned over the dominant M1 FDI hotspot with the handle pointing posterior at 45 degrees to midline. The conditioning stimulator was set to 80% Ultrasound/TMS RMT while the test stimulator was set to 120% Ultrasound/TMS RMT. Ten Ultrasound/TMS stimulations were delivered every 10 seconds for each inter-stimulus interval (ISI) of 1–15 msec. Ultrasound was time-locked to occur 100 ms prior to the conditioning stimulus. The order of the paired pulse ISIs was always ascending from 1 to 15 msec, and this procedure was completed for both tFUS and sham conditions, the order of which was counterbalanced across participants. Stimulation time was approximately 25–30 minutes for each condition, and total experiment time including setup was roughly 2.5 hours.

#### Analysis

Data were first normalized to individual Ultrasound/TMS 120% RMT baseline. The data set was parsed into data windows that represent established ISIs for short intracortical inhibition (SICI) (1–5 msec) and intracortical facilitation (ICF) (10–15 msec)^24^ to test for specific effects of ultrasound on these mechanisms. The intermediate data section (6–9 ISI) was also analyzed separately though these ISIs have not been demonstrated to be specific for either inhibition or facilitation using the paired pulse paradigm. For each data epoch, a separate two-way repeated measures analysis of variance (ANOVA) was conducted with factors MODULATION (sham, ultrasound) and TIME (ISI times). Appropriate post-hoc tests were conducted to investigate any significant effects.

### Experiment 3: Reaction Time

#### Subjects

A total of 28 participants (9 male, 19 female) with a mean age of 22 ± 1.71 years were included in this experiment. Three of the subjects were self-reported left hand dominant.

#### Experimental Procedures

Based upon the above physiological results, we subsequently tested if there is any behavioral result of ultrasound delivered to the primary motor cortex. Specifically, we tested the effect of tFUS on reaction time using a simple stimulus-response task. Participants were required to attend to visual stimulus on a computer screen that consisted of large white block X or O presented on a black background in the middle of the screen. Participants were required to press the space bar on a standard computer keyboard with their dominant thumb when they saw an X and to withhold a response when an O was presented. A total of 100 stimuli were presented at random time intervals between 3–6 seconds with 20% O trials. Ultrasound was time-locked to occur 100 msec prior to the visual stimulus. Participants responded with their thumb while recording EMG activity from the abductor pollicis brevis (APB) which produced a reliable EMG signal during the task. The APB hotspot was determined for every participant using TMS as previously described. We did not use the Ultrasound/TMS coil for this experiment but did employ the same ultrasound transducer with the same parameters as used in the above experiments. The transducer was positioned over the contralateral APB hotspot and held in place with a secure headband. Three conditions were collected. Real and sham neuromodulation were delivered to the APB hotspot and an active control condition was also collected where tFUS was delivered with identical parameters and timing to the vertex of the head as determined using international 10–20 EEG electrode placement site CZ. The order of conditions was randomized across participants.

#### Analysis

Reaction time was defined as the difference between the timing of the onset of the visual stimulus and the timing of the key press as recorded by custom made scripts written in Matlab (Mathworks, Natick, MA, USA). Trials where no key press was recorded or fell outside 1000 msec from stimulus onset or a wrong key was pressed were not included in subsequent analysis. Only trials where the stimulus was an X were included in the reaction time analysis. Trials where the stimulus was an O were included in the catch trial analysis. A correct catch trial response was no key press within 1000 msec of the visual stimulus onset. Any key press within this time window was considered an error and included in the analysis. The average reaction time was calculated from the acceptable trials for each condition (active sham, M1 sham, tFUS) for each participant and this was tested for statistical significance using a one-way repeated measures analysis of variance (ANOVA). Appropriate post-hoc testing was performed for significant main effects. For catch trial data, the number of correct withholding of a key press was expressed as a percentage of total (20) opportunities for each condition for each participant. This data was subjected to a one-way repeated measure analysis of variance (ANOVA) to test for statistical significance between the conditions. Appropriate post-hoc testing was performed for significant main effects.

### Ethics approval and consent to participate

This study was approved by University of Minnesota’s Institutional Review Board and all Participants gave written informed consent to participate. IRB# 1604M86268.

### Availability of data and materials

The datasets used and/or analyzed during the current study are available from the corresponding author on reasonable request.

## Results

### Ultrasound/TMS coil

**Figure 2:**
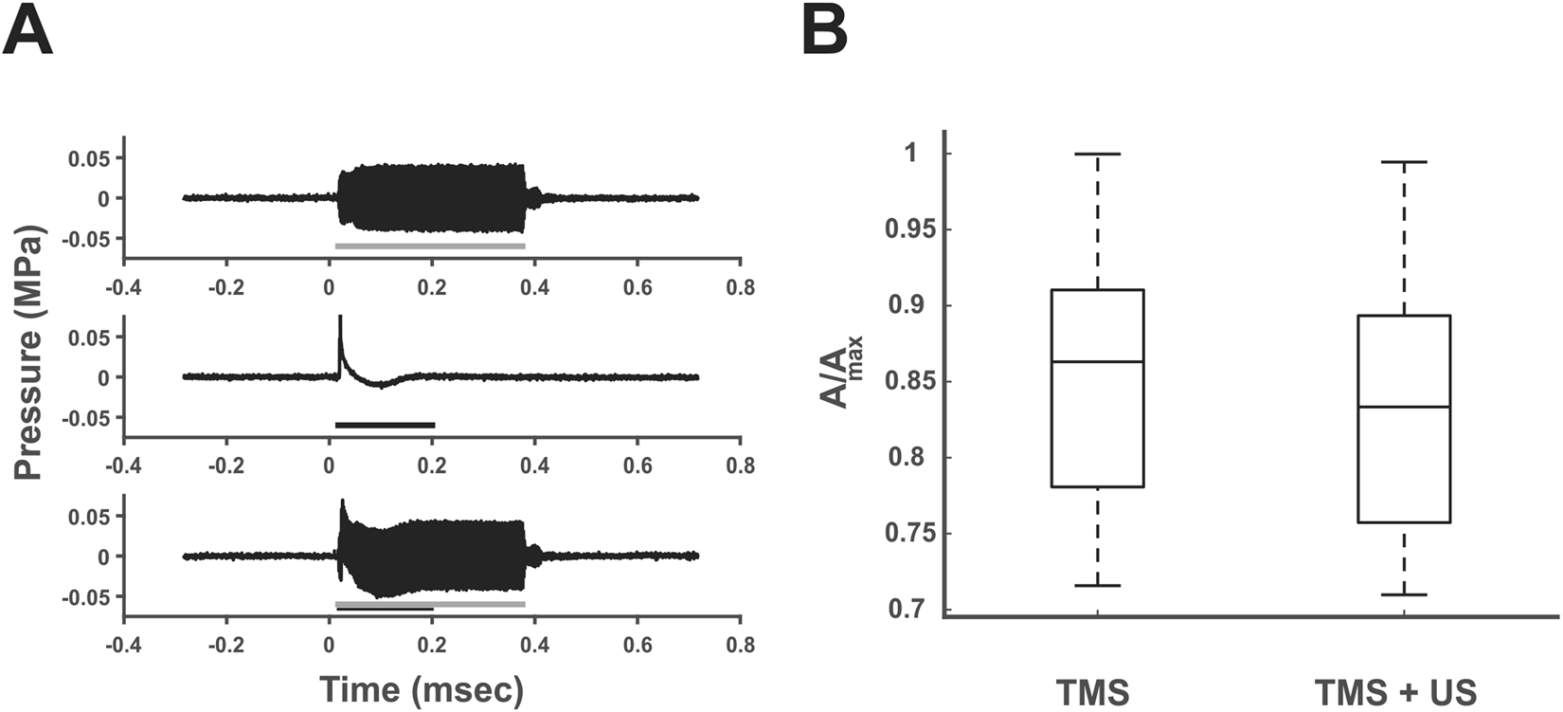
Interaction of ultrasound and TMS. (**A**) Ultrasound field as measured in free water. Grey line represents on time of ultrasound. (Middle) Artifact from single 100% TMS stimulator output pulse. Black line depicts length of pulse artifact. (Bottom) Both ultrasound and TMS are delivered at the same time to determine impact of TMS on the sound field pressure. No effect of the TMS pulse was found on the sound field. The US transducer does not impact the amplitude of the TMS pulse. msec = milliseconds; MPa = megapascals. (**B**) Average magnetic vector potential (A) relative to maximum (Amax) as measured from the plane of the face of the TMS coil without the ultrasound transducer (TMS) and with the ultrasound transducer affixed to the TMS coil (TMS + US). There was no difference in the average magnetic vector potential with the addition of the ultrasound transducer. The central line is the median, the edges of the box represent the 25% and 75% percentiles and the whiskers extend to the most extreme data points.

**Figure 3:**
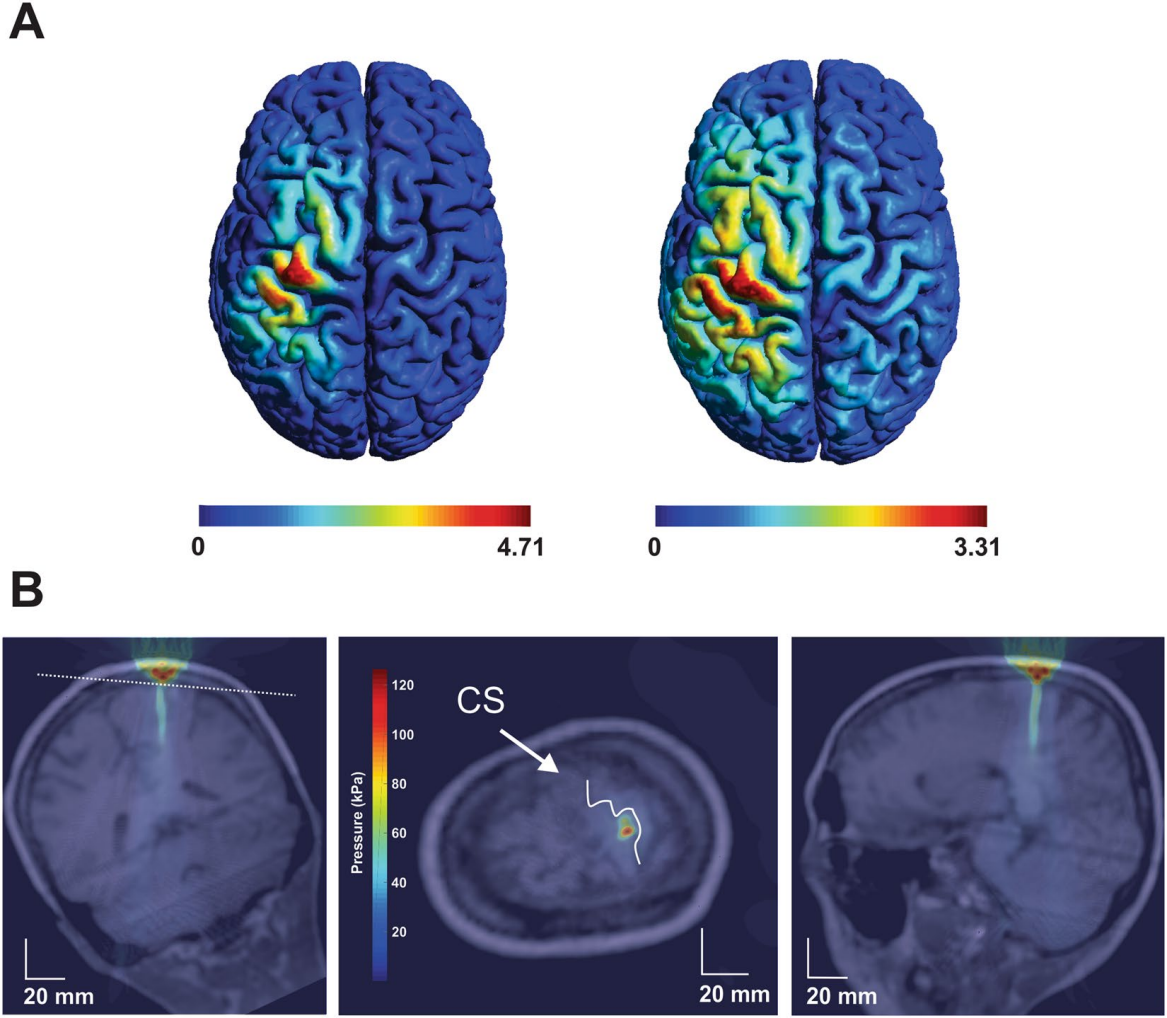
Modelled Ultrasound/TMS field and ultrasound field in the brain. (**A**) Modelled induced electric field in the human brain from the TMS coil without the ultrasound transducer (left) and with the ultrasound transducer (right). (**B**) Acoustic model of the ultrasound waveform positioned over primary motor cortex. CS = central sulcus. Note: Middle figure is transverse section taken through the plane (white dashed line) in the left coronal figure. kPa = kilopascals.

**Figure 4:**
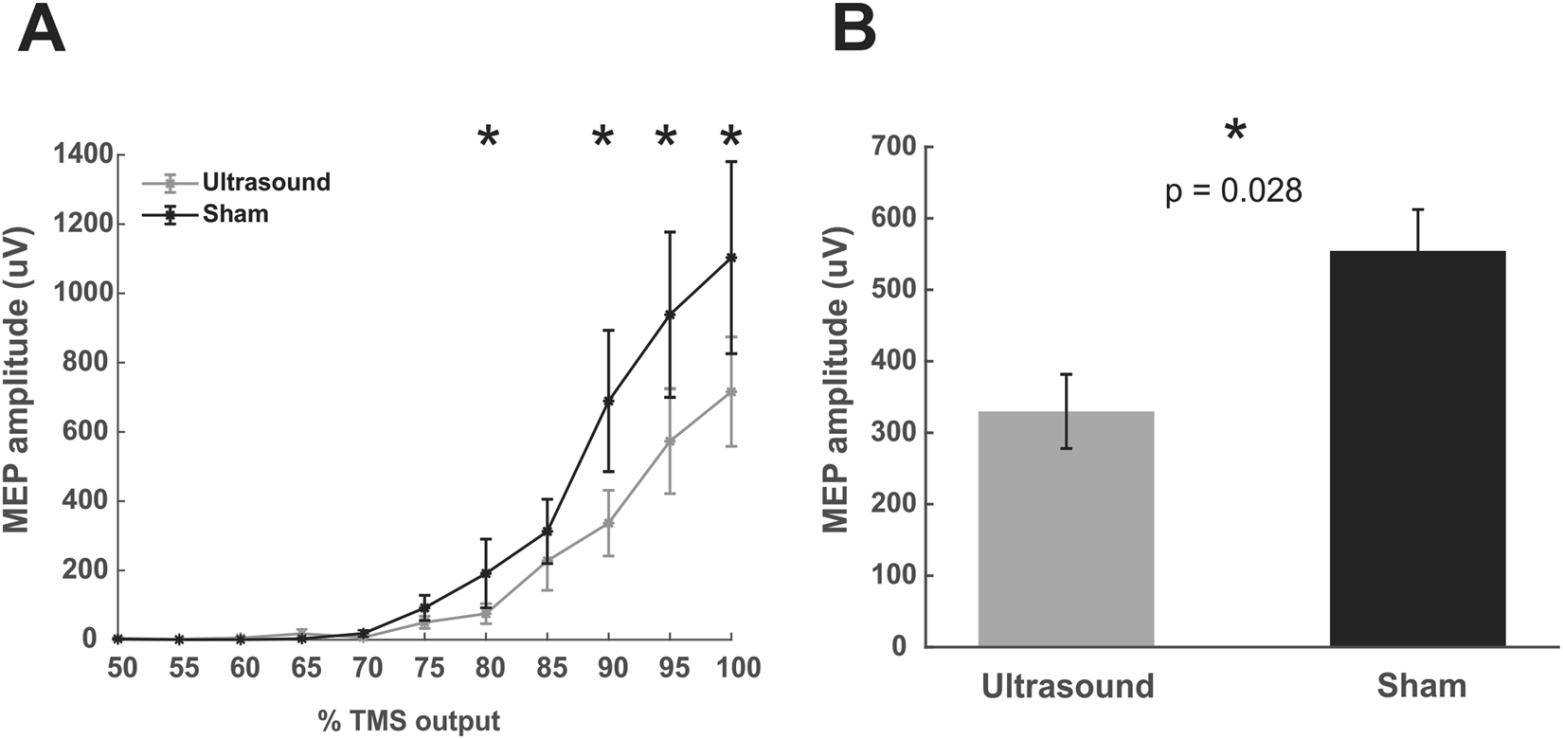
Ultrasound effects on single pulse TMS recruitment curves. (**A**) Group average (N = 12) raw data ± SEM recruitment curves for Ultrasound/TMS (grey) and sham (black) neuromodulation. *Represents p < 0.05 from post hoc testing. (**B**) Main effect of Ultrasound/TMS versus Sham neuromodulation collapsed across stimulator intensities 75–100%. *Represents p = 0.0126.

To ensure no interaction of the energy fields during Ultrasound/TMS we measured the resultant ultrasound pressure profile with and without a concomitant TMS pulse and measured the resultant magnetic vector potential from the TMS coil with and without the ultrasound transducer attached to the face of the TMS coil. We found negligible differences in the amplitude or morphology of the ultrasound waveform with and without a TMS pulse repeated over 100 trials at a TMS stimulator output of 100%. Figure 2A shows a representative trace of the sound field with and without TMS. For 100 TMS pulses with and without ultrasound we found no differences in the magnetic field produced by the TMS coil t(8) = 0.45, p = 0.91 (see Fig. 2B). We repeatedly tested the device at weekly intervals and found no effect on the resultant sound field or the magnetic vector potential with repeated use. We also modelled the resultant electric field in the brain for the TMS coil and the Ultrasound/TMS coil (1.25 cm height offset) to assess the effect of the standoff height of the ultrasound transducer. Increasing the standoff height of the coil 1.25 cm from the head weakens the strength of the induced electric field to roughly 70% (4.71 vs. 3.31 V/cm^2^) of the field produced with the TMS coil directly on the scalp and increases the spread of the induced electric field at the level of the cortical surface holding constant the current fed through the TMS coils (Fig. 3A). The peak induced electrical field in the cortex is still located directly below the center point below the two windings. Thus, by compensating for the loss in electric field strength by increasing the current fed through the coils, the electric field strength at the level of the cortical surface can be maintained above threshold to produce a peripheral muscle response. This is indicated in Fig. 4A where the average % TMS stimulator output to produce a MEP amplitude of ~50 µV was ~75% on average.

### Acoustic modelling

Figure 3B shows an example resultant modelled acoustic field using CT and MRI data directed at the hand knob of the primary motor cortex. These models take into consideration tissue properties that can affect the propagation of ultrasound for neuromodulation^23,25^. It is evident from Fig. 3B that there is good maintenance of the geometry of the sound field and that it is localized to the pre-central gyrus.

### Single pulse recruitment curves

The two-way repeated analysis of variance revealed a main effect of MODULATION (F(1,143) = 6.39, p = 0.0126) where MEP amplitude was significantly attenuated compared to sham (Fig. 4B) and a main effect of TMS INTENSITY (F(5,143) = 9.87, p < 0.001) where MEP amplitudes not surprisingly increase with increasing stimulator output (Fig. 4A). The interaction was not statistically significant (F(5,143) = 0.54, p = 0.74) suggesting that the ultrasound inhibition is not stimulator output dependent. To assess which stimulator outputs contributed to the main effect of ultrasound inhibition, individual repeated measures t-tests were performed for each of the stimulator outputs. This post-hoc analysis revealed stimulator outputs 90%, 95% and 100% were significantly different between Ultrasound/TMS and Sham conditions (p < 0.05) (Fig. 4A).

### Paired-pulse

**Figure 5:**
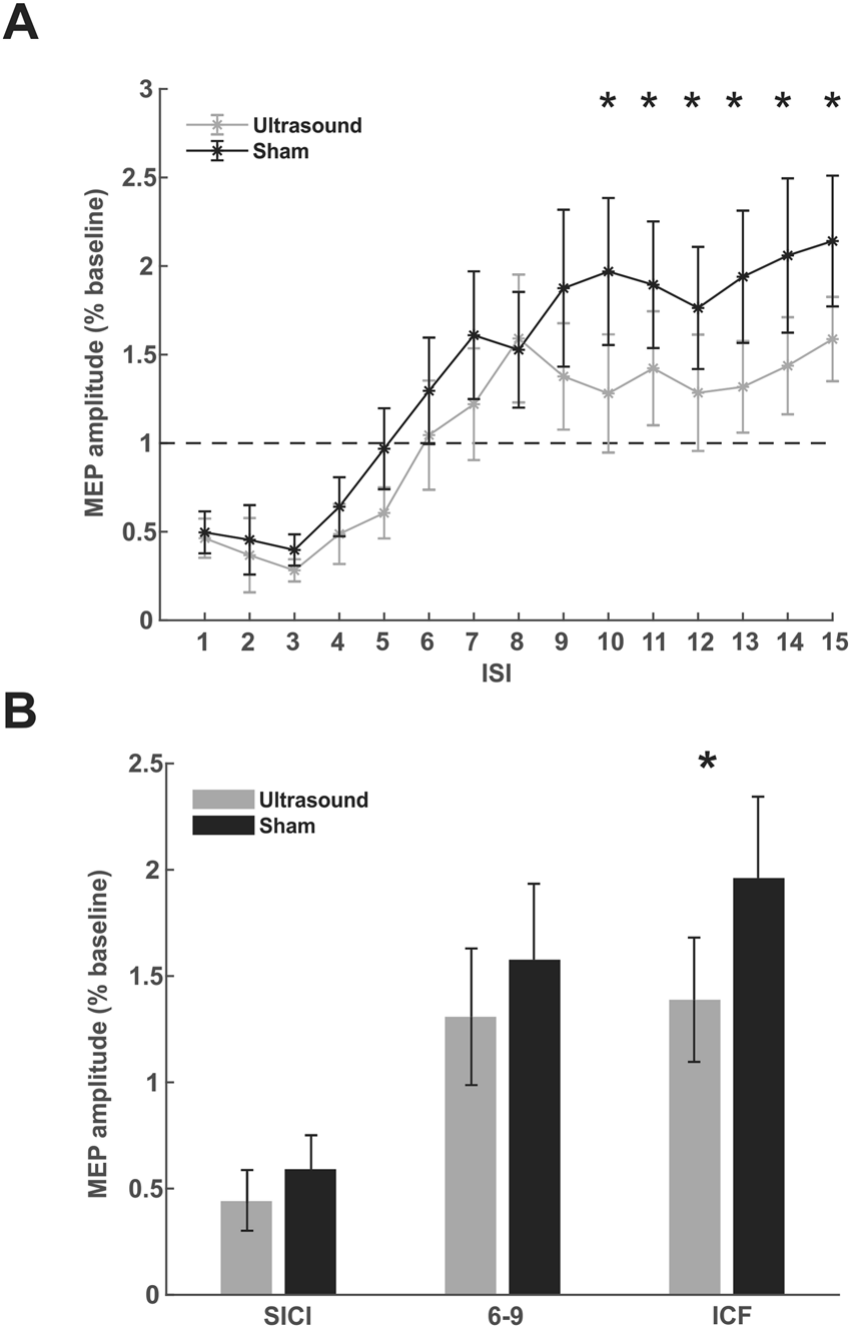
Ultrasound effects on paired-pulse TMS. (**A**) Raw group average (N = 10) paired pulse average motor evoked potential (MEP) amplitude ± SEM for inter-stimulus intervals (ISI) 1–15 milliseconds. Transcranial ultrasonic and magnetic stimulation (Ultrasound/TMS) (grey) and sham (black) neuromodulation. Amplitudes are presented relative to a single pulse baseline (horizontal dashed line). *Represents p < 0.05. (**B**) Averaged normalized MEP amplitude ± SEM collapsed across grouped ISIs known to reflect short intracortical inhibition (SICI) and intracortical facilitation (ICF). *Represents p < 0.05.

Baseline single pulse 120% Ultrasound/TMS RMT MEP amplitude was 340.17 ± 47.27 µV. The two-way repeated measures ANOVA for SICI did not reveal a main effect for TIME F(4, 90) = 2.15, p = 0.08 or MODULATION F(1, 90) = 2.03, p = 0.1574. There was also no significant interaction F(4,90) = 0.29, p = 0.88 (Fig. 5A,B). For the middle epoch consisting of ISIs 6–9 milliseconds the two-way repeated measures ANOVA did not reveal statistically significant effects for TIME F(3, 72) = 0.62, p = 0.60, MODULATION F(1, 72) = 1.11, p = 0.29 or the interaction F(3, 72) = 0.23, p = 0.88 (Fig. 5A,B). For the ICF epoch there was a significant main effect of MODULATION F(1, 108) = 7.56, p = 0.007, no effect of TIME F(5, 108) = 0.21, p = 0.95 and no interaction F(5, 108) = 0.03, p = 0.99 (Fig. 5A,B). The post-hoc Tukey test of the main effect of MODULATION revealed a significant difference for all ISIs 10–15 (p < 0.05) attenuated as compared to sham stimulation (Fig. 5A).

### Reaction Time

**Figure 6:**
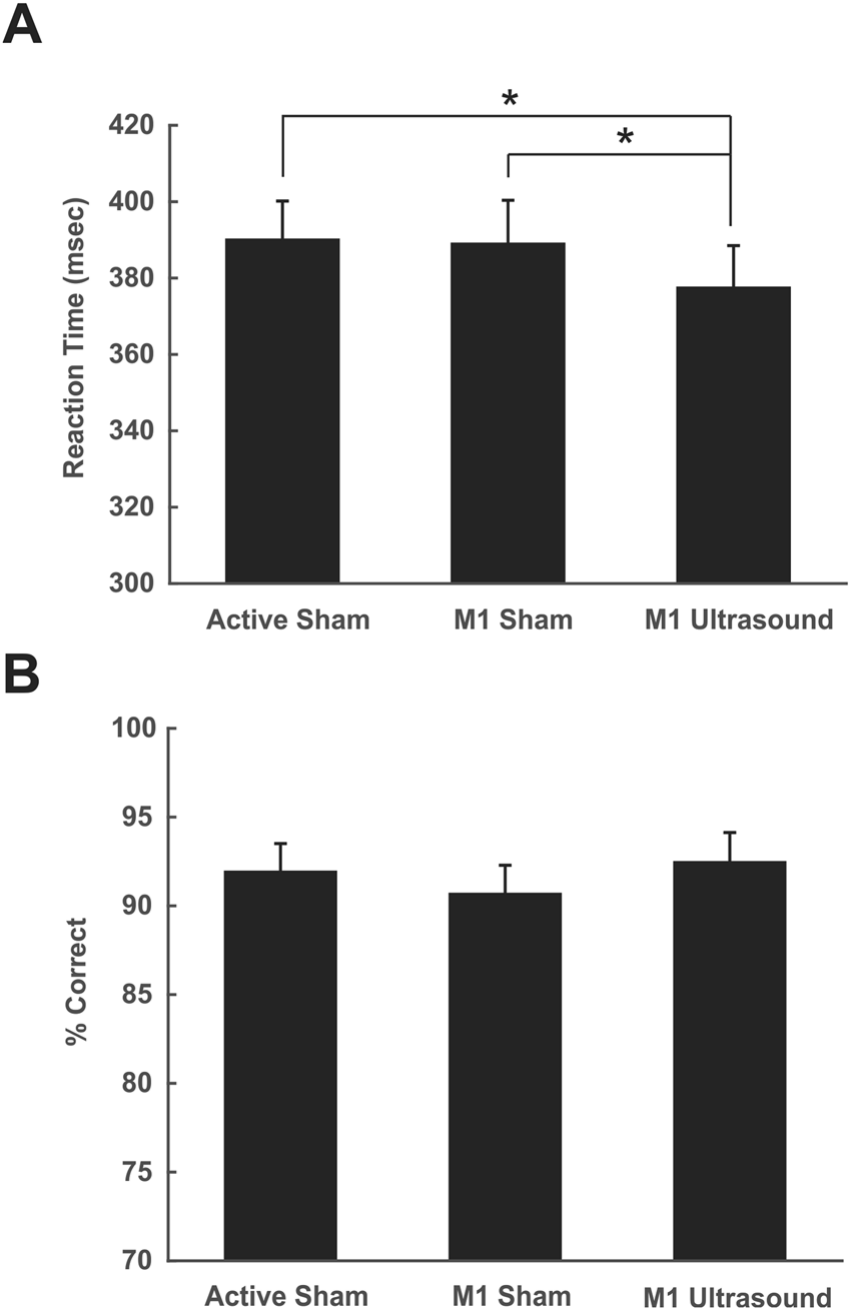
Effect of ultrasound on motor behavior. (**A**) Group average (N = 28) reaction time ± SEM in milliseconds (msec) to a stimulus response task. Active sham represents ultrasound delivered to the vertex. M1 sham represents transducer over primary motor cortex (M1) but no ultrasound. M1 tFUS is transcranial focused ultrasound to primary motor cortex. *Denotes p < 0.05. (**B**) Data from catch trials during the reaction time task. Data is represented as percent correct ± SEM.

It was the purpose of this experiment to test if tFUS to the APB muscle representation during performance of a stimulus response reaction time task performed with the APB affected reaction time. Mean reaction times were 390.1 ± 54.2 msec, 389 ± 61.1 msec and 375.7 ± 58.6 msec for conditions active sham, sham and tFUS respectively. The one-way repeated measures ANOVA revealed a significant effect of condition F(2, 54) = 3.96, p = 0.0248. Post-hoc Tukey test revealed the tFUS condition reaction time to be significantly lower than both the active and passive sham (p < 0.05) (Fig. 6A). In addition to assessing reaction time, we were also able to assess performance on the task using the catch trial data. The percent correct responses to the catch trials were 91.9% ± 8.3%, 90.7% ± 8.5% and 92.5% ± 8.7% for the active sham, sham and tFUS conditions respectively. The one-way repeated measures ANOVA revealed no significant difference between conditions; F(2, 54) = 1.04, p = 0.36 (Fig. 6B).

### Safety

Participants were queried to complete a follow-up Participant Report of Symptoms questionnaire assessing their self-reported experience and tolerance to participation in the ultrasound research and the perceived relation of symptoms to the ultrasound intervention. A total of 20/50 participants (40%) responded to follow-up at various time points out to 15 months post experimentation. No severe symptoms were reported. Mild and moderate symptoms included neck pain, sleepiness, muscle twitches, itchiness and headache. None of these symptoms persisted or got worse.

## Discussion

In the present study we introduce a novel way to assess the effect of ultrasound on motor cortical excitability in humans by pairing it with TMS, thus allowing for quantification of ultrasound effect on measures of the TMS invoked motor evoked potential. We first demonstrate that concentric and concurrent delivery of TMS and ultrasound (Ultrasound/TMS) is feasible and safe in that the energy fields do not interact as tested in this study. Using this pairing, we tested the effect of ultrasound on TMS single pulse recruitment curves, short interval intracortical inhibition, intracortical facilitation as well as on reaction time. Ultrasound did not further attenuate short-interval intracortical inhibition but significantly attenuated intracortical facilitation. Ultrasound directed at the primary motor cortex representation of the muscle performing a simple stimulus response task significantly reduced reaction time. This is the first study in humans to assess the effect of ultrasound to the primary motor cortex and indicates that the ultrasound parameters used here largely conveys an inhibitory effect that confers a behavioral advantage.

### Ultrasound/TMS coil

We found no interaction of the ultrasonic sound field with the magnetic field produced by the TMS coil. We also found no initial or persistent effect of concurrent and concentric ultrasound with TMS on either of the hardware after repeated testing. The current Ultrasound/TMS coil design increases the spread and weakens the induced electric field in the head by roughly 30%, though the area of highest current is still located directly beneath the intersection of the TMS coil windings that is concentric with the ultrasound field, making the Ultrasound/TMS design effective for testing spatially specific motor cortical excitability. This reduction in induced electric field in the head necessitates higher TMS stimulator outputs to reach motor threshold for producing a recordable peripheral motor evoked potential. The TMS stimulator output for achieving motor threshold was in the range of 65–80% of stimulator output. This is an issue though for participants with naturally high motor thresholds, as this may preclude them from participating in experiments that require TMS at significantly greater stimulator output levels with our Ultrasound/TMS offset than their resting motor threshold level as this may exceed the TMS stimulator output. TMS stimulator outputs of 120–130% of resting motor threshold are common for certain TMS protocols and may not always be achievable with the current Ultrasound/TMS configuration. This issue can be minimized with lower profile ultrasound transducers (we used 1.25 cm) or by placing the transducer in plane with the TMS coil windings though TMS wing separation also reduces the induced electric field in the head.

### Biological interaction of ultrasound and TMS

There is some manifestation of electrical effects by ultrasound under certain biological conditions. The ultrasonic vibration potential manifests from ultrasound traversing either a colloidal or ionic suspension and inducing distortion of the charge distributions at the sites of individual particles. The distortion of the charge distribution stems from the particles in suspension having a higher or lower density than that of the surrounding fluid, resulting in a difference in amplitude and phase of particle motion that differs from that of the surrounding fluid during the alternating phases of the acoustic cycle and distorts the charge distribution within the suspension^26^. Relative to the magnitude of neuronal signaling though, the ultrasonic vibration potential is considerably smaller. Vibration potentials at 500 kHz experimentally observed in tissues has amplitudes smaller than 0.02 µV, while vibration potentials in whole blood, which is both colloidal and ionic, has amplitudes on the order of 10 µV^26^. For comparison, the synaptic potentials recorded in a tight-seal cell-attached current clamp of a CA1 pyramidal cell is on the order of tens of millivolts^27^, more than 1,000 times greater than the vibration potential observed in whole blood.

There is the possibility for ultrasound to produce an electric current when in the presence of a magnetic field. The acoustic wave of ultrasound can cause ions to move as outlined above, which in the presence of a magnetic field induces Lorentz forces that create an electrical current^28,29^. The Lorentz force, ${\overrightarrow{F}}_{L}$, of an electric charge, $q$, moving in a magnetic field, $\overrightarrow{B}$, is perpendicular to both the magnetic field and ultrasound velocity of the element, $\overrightarrow{v}$, and is calculated by ${\overrightarrow{F}}_{L}=q\cdot \overrightarrow{v}\times \overrightarrow{B}$, where × is the vector cross product^29^. As applied here, the axis of the propagation of ultrasound and the axis of the magnetic field from TMS are parallel due to location of the ultrasound transducer at the center of the figure of eight TMS coil. As such no Lorentz force is induced during simultaneous tFUS and TMS as the ultrasound velocity vector $\overrightarrow{v}$ and the magnetic field vector $\overrightarrow{B}$ are parallel, which results in a zero cross product vector. Even in the worst case of the TMS magnetic field being oriented perpendicular to the propagation of ultrasound, the induced electric field is relatively low. For a brain tissue conductivity of 0.2 S/m^30^, a peak ultrasound pressure of 0.5 MPa, an acoustic impedance of brain tissue of 1.5 MPa*s/m similar to water, and a peak TMS magnetic field strength of 1 Tesla, the peak induced current due to the Lorentz force is 0.2 Siemens/m * 0.5 MPa/1.5 MPa*s/m * 1 Tesla = 0.067 A/m^2^. This is well below the current density of neuronal activation by TMS (2.5 A/m^2 ^^31^) and still below the peak effects from TDCS/TACS (0.1 A/m^2 ^^32^).

Overall, the electrical effects from either tFUS on its own or in combination with TMS are negligible. Thus, we can begin to examine the issue of other possible interactions of TMS and tFUS by estimating the mechanical and thermal effects of TMS and tFUS individually and then summing them. In our opinion, the thermal risks are the greatest, however the possibility of heating is driven largely by tFUS and dosing and we have previously modeled this and demonstrated heating from the same ultrasound waveform to be less than 0.2 °C in brain^25^. With respect to TMS, tissue heating of the brain by single pulse TMS is very small and estimated to be readily less than 0.1 °C^16^.

For the mechanical interaction between TMS and tFUS, the force of each individually acting on the brain can be calculated and then we can assume that they occur in phase and sum their forces to determine a maximal force of combined effect. Considering the magnetic field gradient along the coil axis, the magnetic translational force on a unit volume of tissue is ${{\bf{F}}}_{m}=\frac{\chi }{2{\mu }_{0}}\nabla ({{\bf{B}}}^{2})$, where $\chi$ is the magnetic susceptibility and ${\mu }_{0}$ is the permeability of free space. For brain tissue, the magnetic permeability across gray and white matter ranges from −9.2 to 8.8 × ${10}^{-6}$^33^, and we shall estimate the magnetic field gradient along the TMS coil axis to be $\nabla {|{\bf{B}}|}^{2}=0.013\,{T}^{2}/{\rm{m}}$^34^. Using a magnetic susceptibility of −10 × 10^−6^ and a volume of 5 mm^3^ (an approximate volume of peak tFUS effects), we estimate a magnetic force of 2.6e-10 N in the brain due to TMS.

Regarding tFUS, the magnitude of the radiation force generated by a propagating plane wave is given by $|{\bf{F}}|=\frac{2\alpha I}{c}$, where *c* is the sound speed, *α* is the attenuation coefficient of the tissue, and *I* is the temporal average intensity. A detailed discussion of the effect of plane wave approximations in acoustic calculations can be found in^35^, but overall the approximation is satisfactory provided a minimum distance from the source. From Legon 2014^1^, the transcranial I_sppa_ is approximately 6 W/cm^2^, therefore the I_spta_ is 2.2 W/cm^2^ (6 W/cm^2^ * 360us * 1 kHz). Using brain acoustic parameters of 1550 m/s for the sound speed and 0.92 Np/m for the attenuation coefficient^25^, the estimate of acoustic radiation force in the brain by tFUS over a similar volume of 5 mm^3^ is approximately 1.3e-7 N.

Due to the difference in magnitude between the TMS and tFUS force estimates, it is readily apparent that the manifestation of forces in the brain is driven by tFUS. Additionally, the heating of brain tissue is driven largely by tFUS as discussed previously. Thus, the physical manifestations of effects, specifically in the thermal and mechanical aspects, are driven largely by tFUS and not TMS, and so no interaction can be expected. As such, concurrent and concentric ultrasound and TMS can effectively be used to probe the effect of ultrasound on motor cortex excitability in humans without inadvertent additional energy from this paired use contributing to the overall effect. This conclusion is underscored by the fact that tFUS is a form of mechanical energy and TMS a form of electrical energy.

### Potential mechanisms of action

It is difficult to assess the exact neuronal population that ultrasound affects in humans non-invasively but the use of the Ultrasound/TMS pairing allows for some inference into the neuronal populations affected by ultrasound as single pulse and paired pulse TMS have been demonstrated to probe different microcircuits in the primary motor cortex^14^. We used a TMS pulse that produced a posterior to anterior induced current in the brain that has been demonstrated to preferentially target more superficial monosynaptic cortico-cortical neuronal connections in cortical layers two and three that project onto corticospinal neurons producing the so-called I-wave as recorded from spinal electrodes^36^. As such, ultrasound may serve to inhibit these populations resulting in the observed MEP inhibition. However, at high TMS stimulator outputs there is the possibility of initiating additional components of the motor micro-circuitry including direct activation of the pyramidal tract neurons. We found increasing attenuation effects with higher stimulation intensities and thus it is possible for ultrasound to exert its inhibitory effects directly on the pyramidal tract neurons in layer five. TMS has limited penetration depth and its effects are mainly limited to more superficial layers of the cortex, though deeper stimulation is possible with increasing stimulator output at the expense of focality^37,38^. Focused ultrasound (depending on the orientation of the gyrus to the axial field) can target deep cortical layers as well as sub-cortical targets^1,20^, so it is possible for ultrasound to directly modulate pyramidal tract neurons. It is unclear if the TMS stimulator intensities we used were high enough to produce this direct activation of the pyramidal tract neurons, though we cannot rule out that ultrasound serves to suppress activity directly at the pyramidal cell axon or cell body. The site of mechanism seems likely to be synaptic given the findings of ^39,40^ that have shown ultrasound to affect ion channels embedded within cellular membranes and that ultrasound does not appear to have an effect on myelinated axons *in vivo*^41^. The fact that we exclusively find inhibition suggests influence of ultrasound on gamma-aminobutyric acid (GABAergic) neurons; however, the specific GABA(A) modulator lorazepam, served to suppress MEP amplitude though only affected motor microcircuitry that produces the late I-waves but not the monosynaptic I wave^42^ and thus, it is unlikely that ultrasound directly affects GABA(A) receptors. The paired-pulse experiment supports our single pulse findings and the contention for ultrasound not to directly affect the circuitry producing the late I-waves. We employed the method of Kujirai *et al*.^24^ and did not find a statistically significant attenuation of MEP amplitude for the known ISIs (1–5 msec) to produce short interval intracortical inhibition. Di Lazzaro *et al*.^43^ specifically showed for the SICI protocol to suppress the late I-waves. Thus, ultrasound’s mechanisms of inhibition likely do not directly act on GABAergic neurons as does lorazepam for example as it increases SICI^42^. There is evidence for some suppression from ultrasound at SICI inter-stimulus intervals (Fig. 5A,B) and we posit that perhaps ultrasound may not be an efficient or as strong a mediator of inhibition that could not further suppress the already well-suppressed state of the motor microcircuitry from the SICI. This could be due to mechano-chemical or mechano-electrical inefficiencies for neural conduction perhaps due to smaller mechanosensitive receptor densities. Previous research has shown that it is difficult to modulate the very robust effect of SICI as an increase in test stimulus amplitude results in an earlier D-wave but the later I-waves are still suppressed^44^.

Despite mostly theoretical proposals of the putative mechanisms of ultrasound^45–47^, the mechanical effects are likely not as robust as direct electro-chemical effects on cellular membranes and/or embedded receptors. There are specific mechanosensitive ion channels in the nervous system^48,49^ that have been demonstrated to be sensitive to ultrasonic perturbation^39,40^ though the proliferation and density of these in the human brain is not well understood. One interesting possibility to help explain the mechanism of ultrasonic inhibition is for influence of the ultrasonic pressure field on glial cells^50^. Astrocytes express mechanosensitive channels and are known to swell in pathological states. This swelling induces depolarization that may be explained by the opening of chloride channels^51^. It may be that the negative pressure produced by ultrasound induces astrocyte swelling and chloride release which may explain the general inhibitory effects we see that mimic GABA as the GABA receptor also selectively conducts chloride through its pore. Indeed, one theoretical framework for describing ultrasound effects posits the negative pressure of the ultrasonic wave to expand the lipid membrane^47^ which is a similar physical mechanism to cellular swelling. This may also explain why we didn’t show statistically significant increases in SICI as this is directly mediated by GABA and the contribution of the astrocytes to this would likely be less robust. In the case where there is ample room for inhibition as in the ICF protocol, ultrasound shows robust inhibition and the astrocyte hypothesis may indeed contribute to this effect as lorazepam does not affect intracortical facilitation^42^, supporting that ultrasound does not directly affect GABA(A) receptors. Unfortunately, the micro-circuitry involved in the ICF protocol is not well understood. ICF does not affect the amplitude or number of descending corticospinal waves despite producing an increase in MEP amplitude suggesting alternate or long-ranging micro-circuitry as compared to single pulse TMS and SICI mechanisms^52^. ICF is considered to be the result of a net facilitation and weaker inhibition from GABA(A) receptors as benzodiazepines decrease ICF though is also likely mediated by N-methyl-D-aspartate (NMDA) receptors as well; as ICF can be decreased by NMDA receptor antagonists^53^.

Ultrasound served to suppress the effect of ICF though not to baseline levels suggesting that, regardless of the specific neuronal population, ultrasound does not serve to totally block excitation but rather its mechanism of inhibition diminishes this effect perhaps by producing a more general state of inhibition that renders the neuronal circuitry responsible for ICF less receptive to excitation.

As concerns the paired-pulse protocol, ultrasound was delivered overlapping with both the conditioning stimulus and the test stimulus. Seeing as ultrasound has an effect on single-pulse TMS (Fig. 4A,B) it is possible that ultrasound also has an effect on the conditioning stimulus and its ability to activate the low threshold inhibitory neurons that suppress the excitatory inputs activated by the test stimulus. We do not believe this to be the case as even under the ultrasound condition there is similar robust inhibition for SICI demonstrating that the conditioning stimulus was not rendered impotent and indeed resulted in robust inhibition. The conditioning stimulus also was effective in the ICF condition as the test stimulus MEP amplitudes were facilitated compared to baseline only that there was an attenuation of this effect with the ultrasound condition. The fact that we see a differential effect between SICI and ICF suggests that the ultrasound effect is not an effect solely on the circuitry activated by the conditioning stimulus but reflects an effect on the microcircuitry probed by the test stimulus and that this is exclusive to that involved in ICF. Furthermore, based upon the results from the single pulse study (Fig. 4A,B) there is no observable effect at lower single pulse TMS outputs that would be commensurate with the conditioning stimulus amplitude. Further research could look to initiate ultrasound between the conditioning stimulus and the test stimulus to precisely isolate the effect of ultrasound to the circuitry the test stimulus probes.

Additionally, we did not find ultrasound to affect MEP threshold. This is perhaps not too surprising as an increase in TMS output, while increasing evoked potentials, does not affect the threshold for activation. This suggests that the neural populations activated by posterior to anterior TMS induced electric fields have a constant threshold that is unaffected by altered synaptic activity^52^ and ultrasound did not affect this property of the motor micro-circuitry.

### Comparison to motor cortex animal studies

There are numerous reports in mice^2,3,54^ as well as in rats^4,5^, rabbit^6^ and sheep^7^ for ultrasound directed at the motor cortex to result in peripheral EMG activity. This de facto represents pyramidal tract neuron excitation. At no point during any of the experiments conducted here did we find ultrasound to spontaneously elicit EMG activity. It is not currently clear why this is possible in animal models and not here, but may be due to the parameters used, the size of the skull relative to the pressure field and/or perhaps due to other indirect considerations^55^. Specific parameters have been titrated in these small animal preparations that show preference for either excitation or inhibition^3,4^ though the waveform we employed has previously shown only inhibition^1,13^ and has not been explicitly tested in small animal preparations. It is possible with changes in the duty cycle, amplitude or duration, excitation could be achieved in human motor cortex. Small changes in different parameters (also based on preparation) also may explain the difficulty in producing robust excitation^5,56^. Of more importance, may be the effect of skull size or cranial volume relative to the ultrasound pressure field. In recent work, we showed how skull size impacts the intracranial pressure field such that smaller skulls interact with the main acoustic beam more^23^. Previous numerical simulations of experimental setups with rats have found substantial intracranial pressure increases in rat skulls compared to the corresponding transducer’s free-field pressures^5^. There is greater wave confinement in smaller skulls that will result in stronger and more numerous interactions, including standing waves which could explain motor responses in smaller animals. Indeed, King *et al*.^3^ demonstrated ultrasound activation to be a function of the product of duration and amplitude and Kubanek *et al*.^39^ showed the ultrasound effect to be a function of pressure. Thus, it is possible that our waveform combined with our relatively low intracranial modeled pressure of ~120 kPa is not sufficient for neural excitation.

### Behaviour

We previously found ultrasound with identical parameters as used here to inhibit activity of the primary somatosensory cortex that also resulted in a behavioral improvement in tactile sensitivity^1^. Here, we also found a behavioral advantage to this ultrasonic neuromodulation concomitant with physiological inhibition. We are confident this behavioral effect is not due to extraneous attentional or potential cueing mechanisms from the ultrasound as we employed an active sham in addition to the passive sham that demonstrated no effect on reaction time. In addition, the data on the catch trials did not show any differences, again demonstrating the effect was not due to attentional factors. It may be paradoxical at first thought that physiological inhibition confers a behavioral advantage but it is a common finding for the balance of excitation and inhibition (or loss of inhibitory tone) to underlie some behaviors^57^ and neurological conditions such as schizophrenia^58^. There is evidence in the human motor cortex for inhibition that is necessary for fractionated finger movement that has been demonstrated to be dysfunctional in disease states such as focal dystonia^59^. Perhaps ultrasound provides for a mechanism to sharpen the inputs to the specific finger representation that aids performance. Single finger movements are never completed isolated contractions of one muscle but need simultaneous control of the entire hand and forearm involving the contraction of many muscles acting on different fingers and joints^60,61^. As such, perhaps ultrasound serves to suppress the inputs of the other muscle representations onto the effector, increasing the fidelity of the response signal to this effector and reducing transmission and execution time. These mechanisms are largely mediated through GABAergic transmission, that from our single and paired pulse experiments ultrasound looks to at least indirectly affect. The ultrasound field has a small ~3–4 millimeter diameter that is commensurate with the size of individual finger representations within the motor homunculus^62^ and thus it would be feasible to test the excitability of individual muscle representations during specific movements to help better understand how ultrasound contributes to motor behavioural changes.

## Conclusions

We present here a new method to probe the effect of ultrasound on human motor cortical excitability. The Ultrasound/TMS device allows for a quantifiable method to assess the effect of ultrasound on human primary motor cortex excitability as direct elicitation of motor evoked potentials from ultrasound has not yet been demonstrated. This method can be exploited to provide for detailed exploration of ultrasound parameters and duration of effect in humans that could not previously been achieved. The Ultrasound/TMS method can help progress human brain mapping efforts and eventual application of low intensity focused ultrasound to clinical populations for specific targeting and assessment of brain pathology.
